# Is “cellular senescence” a misnomer?

**DOI:** 10.1007/s11357-022-00652-x

**Published:** 2022-09-07

**Authors:** David Gems, Carina C. Kern

**Affiliations:** Institute of Healthy Ageing, and Research Department of Genetics, Evolution and Environment, University College London, London, WC1E 6BT UK

**Keywords:** Aging, Cellular senescence, Fibroblast, Osteoarthritis, Remodeling-associated secretory phenotype (RASP), Remodeling activation

## Abstract

One of the most striking findings in biogerontology in the 2010s was the demonstration that elimination of senescent cells delays many late-life diseases and extends lifespan in mice. This implied that accumulation of senescent cells promotes late-life diseases, particularly through action of senescent cell secretions (the senescence-associated secretory phenotype, or SASP). But what exactly is a senescent cell? Subsequent to the initial characterization of cellular senescence, it became clear that, prior to aging, this phenomenon is in fact adaptive. It supports tissue remodeling functions in a variety of contexts, including embryogenesis, parturition, and acute inflammatory processes that restore normal tissue architecture and function, such as wound healing, tissue repair after infection, and amphibian limb regeneration. In these contexts, such cells are normal and healthy and not in any way senescent in the true sense of the word, as originally meant by Hayflick. Thus, it is misleading to refer to them as “senescent.” Similarly, the common assertion that senescent cells accumulate with age due to stress and DNA damage is no longer safe, particularly given their role in inflammation—a process that becomes persistent in later life. We therefore suggest that it would be useful to update some terminology, to bring it into line with contemporary understanding, and to avoid future confusion. To open a discussion of this issue, we propose replacing the term *cellular senescence* with *remodeling activation*, and SASP with *RASP* (remodeling-associated secretory phenotype).

## Introduction 

As anyone who has ever marked student essays on aging will know, the terms *senescence* and *cellular senescence* are easily confused. Senescence refers to age-related deterioration (aging in other words), while cellular senescence is a particular type of change that can occur in some cell types in vertebrates, typically involving entry into a hypertrophic, hypersecretory state [1].

How cellular senescence is understood has changed over the years. When it was originally observed that after many rounds of subculture, cultured fibroblasts lose the ability to divide and then seemingly die off, Leonard Hayflick deduced that such replicative exhaustion involves some form of cellular aging (senescence), or *replicative senescence* [2]. Subsequent work established that after ceasing to divide fibroblasts do not in fact die off, but can be maintained in culture for years [3]. Moreover, such cells become enlarged and secrete a complex cocktail of growth factors, cytokines, chemokines, extracellular matrix (ECM) components, and proteases into the media—the senescence-associated secretory phenotype (SASP) [4, 5]. In a subtle shift of meaning, cells in this arrested, hypertrophic and hypersecretory state became known as *senescent cells* and their entry into this state as *cellular senescence*. It was then demonstrated that during aging, senescent cells accumulate within tissues and promote disease, particularly due to effects of the SASP [1, 6, 7].

Here, we argue that over the course of the last decade studies of the biology of cellular senescence in vivo have so transformed our understanding that the term “cellular senescence” has ceased to be accurate. What were initially viewed as damaged and dysfunctional cells are now understood to be, in many contexts, normal, healthy cells that contribute to normal development, and to tissue homeostasis and remodeling during adulthood [8–12]. An emerging perspective is that it is pathogenic action of such developmental functions in later life that promotes late-life disease [13]. Thus, it may be argued that, thanks to recent advances, the term “cellular senescence” has become obsolete and misleading. Like children’s shoes, it has been outgrown. We suggest that the time has come to replace it.

## Stochastic and programmatic causes of cellular senescence

Cellular senescence is often presented as a consequence of stress and damage. Consistent with this, DNA damage can trigger cellular senescence. Early studies established that replicative senescence in cultured human cells is attributable to shortening of telomeres [14]. Importantly, DNA damage (including telomere shortening) triggers cellular senescence through tumor suppressors such as p53, p21, and p16, and oncogene activation is sufficient to cause cellular senescence (oncogene-induced cellular senescence, or OIS) [15]. Thus, damage-induced cellular senescence provides a means to kick potentially cancerous cells out of the cell cycle, thereby reducing cancer risk [1]. However, as a side effect, senescent cells accumulate in later life, and actually promote cancer development through influence of the SASP; therefore cellular senescence promotes fitness in early life but causes pathology in later life, an example of antagonistic pleiotropy [16, 17]. Why senescent cells should be so perversely destructive was initially a mystery.

That DNA damage causes cellular senescence also chimed with the long-standing belief that aging is predominantly a function of molecular damage and somatic maintenance. This may have reinforced the now widespread view that senescent cells can be understood as damaged cells, and therefore senescent in the original meaning of the word (since aging is damage). However, findings over the last two decades have led to doubts about how important molecular damage is as a cause of aging, and to the emergence of an alternative paradigm. Drawing on diverse experimental findings, the programmatic (or hyperfunction) theory argues that mechanisms of aging are to a large extent driven by wild-type developmental processes that cause pathogenic changes in the composition and properties of tissues and organs [18–22]. Although specified by the wild-type genome, such deteriorative changes are not adaptive: though programmed in the mechanistic sense they are not programmed in the adaptive sense [23], and may therefore be described as *quasi-programmed* [18] or *programmatic* [22]. Here senescence results not from *loss* of function resulting from damage, but rather the opposite: *hyper*-function [18]. How such programmatic changes might arise from the evolutionary process, including the role of antagonistic pleiotropy, is discussed further elsewhere [17, 22, 24].

Mikhail Blagosklonny originally developed the concept of quasi-programmed hyperfunction after observing that cellular senescence in cultured fibroblasts can be induced by simultaneously blocking the cell cycle and promoting growth, without any involvement of damage [18, 25]. Moreover, cultured senescent fibroblasts can trigger cellular senescence in their non-senescent neighbors (paracrine senescence) [26], again implying induction by damage-independent mechanisms. Thus, cellular senescence in vitro can be induced by both damage-dependent and damage-independent mechanisms.

But what about the situation in vivo? Here the capacity for DNA damage to induce cellular senescence has been amply demonstrated [27], particularly by increased levels of cellular senescence seen in mice with artificially high levels of DNA damage [28–30]. Yet, the origins of the senescent cells that accumulate in vivo during normal (wild-type) aging remain poorly understood [31]; thus, the relative importance of damage accumulation vs programmatic mechanisms in late-life increases in senescent cells remains an open question. If programmatic mechanisms contribute significantly to cellular senescence in vivo, what are the adaptive programs from which the harmful quasi-programs later arise?

## Cellular senescence can result from damage or developmental change

This brings us back to the question of why senescent cells in vivo are so actively destructive. Why would natural selection favor the production of highly pathogenic SASP? Here, it is helpful to recall that the cell type most often used to study cellular senescence is the fibroblast. The main function of fibroblasts is in tissue development and maintenance, including repair of tissue injury [32], particularly through synthesis and remodeling of the ECM in which cells are embedded, and provision of positional information [33]. Thus, fibroblasts assure somatic maintenance, but more at the tissue level than the molecular level, i.e., the type of somatic maintenance involved is more developmental and architectural than molecular. Tissue injury activates fibroblasts’ tissue repair activity, rather than damaging the fibroblasts themselves (though stress to individual fibroblasts may trigger their repair responses).

Many of the significant advances relating to cellular senescence have arisen from research led by Judith Campisi, at the Lawrence Berkeley National Laboratory and then the Buck Institute for Research on Aging. A major discovery by her team, reported in 2014, was that large numbers of senescent fibroblasts appear transiently in skin lesions, where they play an active role in the wound healing process [34]. The proteases of the SASP aid in the process of debriding the wound, the clearing out of damaged matter prior to replacing it with new tissue. The SASP growth factors and cytokines help promote formation of new tissue, and SASP chemokines recruit other cell types (particularly macrophages) to the task of wound healing. Thus the hypertrophic and hypersecretory nature of senescent cells, rather than being some sort of aging-induced cellular derangement, reflects their useful role in tissue remodeling.

It is by now clear that senescent cells play a similar remodeling role in many contexts [13], including repairing tissue injury not only in the skin, but also the liver, heart, muscle, lung, and eye [9]; contributing to morphogenesis during embryonic development [35]; triggering parturition [36]; and even promoting organ and limb regeneration in teleost fish (e.g., zebrafish) and amphibians (e.g., salamanders) [11, 12].

The healing of skin wounds is just one element of the wider spectrum of inflammatory processes that effect tissue remodeling and repair. Notably, progressive increases with age in levels of inflammation throughout the body contribute to many diseases of aging, diseases in which accumulation of senescent cells is also observed. These include atherosclerosis, chronic kidney disease, idiopathic pulmonary fibrosis, osteoarthritis, osteoporosis, diabetes, obesity, and sarcopenia [10]. Age increases in senescent cell number are symptomatic of late-life, chronic inflammation, which can be understood as an inflammatory quasi-program arising from futile and persistent activation of adaptive, acute inflammation processes that promote tissue repair [9, 10, 37, 38]—processes of which senescent cells are a part.

Arguably, the discovery of this remodeling role rather pulls the rug out from under the concept of cellular senescence. It clearly makes little sense to refer to fibroblasts engaged in wound healing or, particularly, in embryonic development as “senescent”—as in the strange construction *developmental senescence* [13]. How, then, might “cellular senescence” be redefined to avoid such confusion? One possible way is to draw a distinction between two forms of cellular senescence: *adaptive* (as in fibroblasts aiding in wound healing) and *non-adaptive* (such as that resulting from DNA damage) [22]. Arguably though, this does not cut deep enough. The difficulty remains that the construction *adaptive cellular senescence* is still problematic, given that such cells are not in any way senescent in the proper sense of the word. *Adaptive cellular senescence* is also something of an oxymoron, given that aging is non-adaptive according to evolutionary theory [17, 39].

## Senescent cells are remodeling cells

**Fig. 1:**
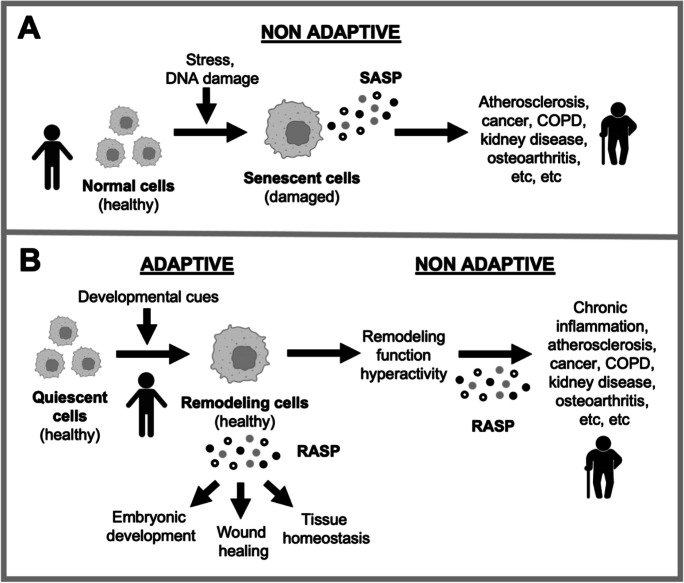
Rethinking cellular senescence. **A** Traditional conception of cellular senescence. Damage to cells causes exit from the cell cycle and abnormal behavior (hypertrophy, hypersecretion) that contributes to aging pathology, particularly through action of the SASP (senescence-associated secretory phenotype). **B** Revised view. Remodeling activation (an alternative descriptor for cellular senescence) supports multiple tissue remodeling functions, including embryogenesis, acute inflammation (including wound healing) and tissue homeostasis, particularly through action of the RASP (repair-associated secretory phenotype). Such cells are healthy and useful, and not senescent in any true sense of the word. In later life, futile remodeling quasi-programs, including chronic sterile inflammation, contribute to aging pathology [9]. Here, RASP becomes pathogenic, e.g., due to chronic secretion. Developmental cues include those directing restoration of tissue architecture after injury [33]

If it is not possible to fix the term cellular senescence by adding qualifiers then perhaps it would be worth considering replacing it altogether? Here, for discussion, we offer some suggestions (and others may have better ones). Given that adaptive cellular senescence involves activation into a tissue remodeling state, we suggest the replacement term *remodeling activation*. Adaptive “senescent” cells may then be referred to as *remodeling cells* (Fig. 1). For remodeling cells, the term senescence-associated secretory phenotype (SASP) is, likewise, unsuitable and may be referred to instead as *remodeling-associated secretory phenotype* (*RASP*). Similarly, paracrine senescence may be referred to as *paracrine remodeling activation*, and the distinctly preposterous term *developmental senescence* replaced with *developmental remodeling activation*.

But, arguably, there is a still deeper cut to be made here, relating to *non-adaptive cellular senescence*. Does it actually exist (i.e., does it involve senescence in the true meaning of the word)? Cells can respond to DNA damage by blocking the cell cycle, as a defense against cancer [1]. In growth-stimulated fibroblasts, blocking the cell cycle can trigger differentiation into the hypertrophic, hypersecretory remodeling state [25]. Part of the remodeling program is production of RASP signals to the immune system to trigger removal of the remodeling cell once its work is complete [40]. Along with blocking the cell cycle, the tumor suppressor program repurposes this component of RASP function to eliminate potential cancer cells. Here, the “cellular senescence” triggered by tumor suppressor programs is adaptive; again, such cells are not senescent in the true sense of the word. To drive this point home, another way that tumor suppressor programs get rid of potentially cancerous cells is by inducing apoptosis; cells dying in this way are not senescent either, in the true sense. More precisely, the tumor suppressor program triggers RASP-directed cell clearance. Thus, one may refer to *oncogene-induced remodeling activation* (*OIRA*, cf OIS, oncogene-induced senescence).

The existence of RASP-directed cell clearance provides a clue to the possible significance of another feature of remodeling cells: their resistance to apoptosis. A hypothetical possibility is that in some contexts in which remodeling cells are active (e.g., wound healing), triggers of apoptosis (e.g., growth signal hyperactivity, DNA damage) may be present. Hence, apoptosis needs to be switched off to enable remodeling cells to do their work. The increased cancer risk due to DNA damage is not an issue here, since remodeling cells are normally destroyed once their job is done.

To reiterate a key point, when Hayflick suggested that fibroblasts in exhausted tissue cultures were senescent, he meant this in the true sense of the word: such a limit to proliferation could “bear directly upon problems of aging, or more precisely, ‘senescence’” [2]. In other words, the conjecture was that cultured fibroblasts eventually stop dividing and die as the result of *aging* [41]. Yet, since these pioneering studies, understanding of this cell biology has transformed to a degree that by now use of the label “senescent” is more traditional than meaningful. Cells active in remodeling processes, or committing suicide via RASP-induced immune clearance are not senescent in the proper sense. The term “senescent” here is like a label saying “banana” attached to an orange.

## Cellular senescence vs remodeling activation as a cause of osteoarthritis

A concern is that confusion created by the cellular senescence concept might have, at times, confounded attempts to understand aging. A possible example of this relates to the disease osteoarthritis (OA). A recent hypothesis is that OA is promoted by accumulation in articular joints of chondrocytes that have become senescent, as the result of stress-induced damage (including DNA damage) [42]. Supporting this account, chondrocytes from articular cartilage in older people show increased levels of two markers of cellular senescence: senescence-associated -β-galactosidase (SA-β-Gal) and p16^INK4a^ (p16) [43, 44]. However, both markers are also characteristic of developmental remodeling activation, as shown in fibroblasts involved in wound healing [34]. Thus, it is possible that such marker-positive chondrocytes are engaged in tissue remodeling.

We recently developed an account of the role of activated remodeling chondrocytes in the wider pathophysiology of OA (D. Gems and C.C. Kern, in preparation). This incorporates an earlier developmental model of OA etiology [45], which is as follows. Two types of cartilage can be distinguished: temporary cartilage which is an intermediate step in the process of bone development, and permanent cartilage which remains as cartilage, as in the hyaline cartilage that lines articular joints. Bone development involves a program in which chondrocytes synthesize first temporary cartilage, and then bone. To generate permanent cartilage, the bone development program is effectively blocked in mid-stream, through action of TGF-β [46, 47]. According to the theory, during aging the TGF-β block becomes disengaged, causing the chondrogenic program to roll on into bone formation [45], i.e., for program to run on into quasi-program. This could account for the bone overgrowth that characterizes OA (including bone spur formation). Consistent with this, blocking TGF-β signaling in mice leads to high levels of bone-generating chondrocytes in their articular cartilage [46].

In the normal process of bone development from cartilage (endochondral bone formation), activated chondrocytes become hypertrophic, increasing 10–15-fold in volume [48], and hypersecretory, with a RASP. The latter includes collagen X, which is used as a marker to identify bone-forming chondrocytes. Collagen X is not expressed in healthy adult articular cartilage, but appears in arthritic joints where new bone growth is taking place [45, 49, 50]. Activated, hypertrophic chondrocytes also promote bone fracture repair [51], just as activated remodeling fibroblasts promote wound healing [34]. Taken together, this suggests that the senescence marker-expressing chondrocytes associated with OA may in fact be bone-generating, hypertrophic cells, i.e., that such chondrocytes are not damaged, but rather are normal and healthy—though hyperfunctional relative to what is optimal for tissue health [52] (Fig. 1b).

Another area of understanding that may have been obfuscated by the concept of damaged, harmful senescent cells accumulating with age involves the relationship between cellular senescence and late-life inflammation. Cellular senescence is sometimes viewed as a cause of inflammation, such that removing senescent cells reduces inflammation and facilitates repair [38]. More precisely, using fibroblasts as an example: prior to aging, fibroblasts in the activated remodeling state are *part* of the repair process that is acute inflammation [34]. In this process, fibroblasts act in concert with macrophages [12], a similarly hypertrophic and hypersecretory cell type [53].

Notably, pro-inflammatory M1 polarized macrophages present in the earlier stages of acute inflammation can also express SA-β-Gal and p16^INK4a^, and such macrophages increase in number during aging [54]. Their elimination could in principle have contributed to the life-extending effects of deletion of p16-expressing cells [6, 54, 55]. Notably, like senescent cells, macrophage action contributes to diverse diseases of aging, including cancer, osteoarthritis, atherosclerosis, diet-induced insulin resistance, and fibrosis [56]. Thus, in later life, activated remodeling fibroblasts are not so much a cause of chronic inflammation as a component of it, as part of an inflammatory quasi-program: broadly speaking, of acute inflammation repair processes which fail to resolve [57].

Similarly, in later life, microglia of the brain proliferate and activate, leading to neuroinflammation and, in turn, neurodegeneration. They also increasingly express SA-β-Gal and p16^INK4a^, which has been interpreted as cellular senescence [58–60]. An alternative possibility is that this reflects inflammatory remodeling activation in these tissue resident macrophages.

In a good illustration of the pathogenic developmental role of remodeling activation, “senescent” cells play a critical role in the enamel knot, a transient embryonic signaling hub that directs tooth morphogenesis. Similar cells appear in a type of benign brain tumor called a craniopharyngioma, in which odontogenetic programs are activated, occasionally leading to fully formed teeth [61, 62].

## Reconceiving senotherapy

The ground-breaking discovery that elimination of p16-positive cells retards development of multiple diseases of aging in mice [6, 55] provided proof for the principle of *senotherapy*. The emerging perspective was that damaged, senescent cells that accumulate with age are a pathogenic agent whose pharmacological removal or inhibition should improve late-life health [63]. But arguments presented here paint a somewhat different picture. They suggest that p16-positive cells that contribute to pathology often do so by acting within late-life quasi-programs arising from beneficial remodeling programs operative earlier in life, as for example when destructive chronic inflammation develops from benign, reparative acute inflammation [9]. Thus, another way of looking at senotherapy is that it works by suppressing hyperfunctional quasi-programs.

Such a reconception of senotherapy is not an argument against its efficacy. For example, returning to osteoarthritis, injury-induced OA in mice can be reduced by eliminating p16-positive cells [64]. As argued here, such cells may be hypertrophic bone-forming chondrocytes; thus, the benefit from their removal is consistent with the osteogenic quasi-program model of OA. However, given that the hypertrophic chondrocytes that promote OA are not senescent in the true sense of the word, it is inaccurate to refer to as “senotherapy” their removal in order to treat OA. As an alternative term, one could say that eliminating p16-positive cells in older individuals is a form of *anti-hyperfunction therapy*, since it is when remodeling cells become hyperfunctional that they become pathogenic.

But anti-hyperfunction therapy describes a wider category of intervention. For example, chondrocyte hypertrophy is promoted by mTOR (mechanistic target of rapamycin) signaling [65], as is that of remodeling fibroblasts [66]. Moreover, mTOR also promotes bone growth, which can be inhibited by rapamycin [65, 67], suggesting that rapamycin should inhibit quasi-programmed bone formation, i.e., inhibit OA. In fact, both rapamycin and cartilage-specific removal of mTOR inhibit OA in mice [68–71]. This suggests that mTOR inhibitors such as rapamycin might be able to inhibit the development of OA in humans, particularly if applied when quasi-programmed endochondral bone formation is actively taking place. In conclusion, reconceiving senotherapy as anti-hyperfunction therapy, with the better understanding that this brings can, arguably, enable better therapeutic design.

## Concluding remarks

The meaning of the term *cellular senescence* has shifted over the years as its biology has become better understood. There is nothing wrong in itself with a word taking on a new, additional meaning, as long as it is not a source of confusion. But such clear differentiation between meanings does not exist in the case of cellular senescence. Scientific terminology needs to be precise. Yet, perhaps the most important consideration when proposing new scientific terminology is utility, and the fostering of progress in understanding. Does the new terminology usefully capture an idea or describe phenomena in a way that enables clearer thought and discussion? And is the long-term benefit worth the short-term cost of confusion caused by the changeover?

We have argued here that the terminology of *cellular senescence* (including *SASP*, *paracrine senescence*, and *senotherapy*) has been outgrown by advances within the field, such that they risk becoming obstacles to scientific progress. A comparable problem arose previously from a conception of stem cells that was based on hematopoietic stem cells. This subsequently proved inapplicable to solid tissue stem cells, motivating a redefinition of the concept that draws more on function than on cellular properties [72]. We hope that the ideas presented here will encourage discussion of this issue in the aging research community. It may be appropriate to convene a meeting of researchers in the field to discuss the matter.

That the concerted action of “senescent” cells and macrophages in tissue remodeling is evolutionarily ancient, seen even in fish and amphibians [11, 12], suggests that this is the original, ancestral role of such cells. Arguably, the pathogenic action of “senescent” cells in later life is best understood as an element of wider tissue-remodeling programs that become hyperactivated in later life (as in persistent inflammation) [13]. Here, as in iatrogenic diseases, a response to alleviate pathology makes it worse, leading to such conditions as fibrosis, which contributes to almost half of all deaths in the developed world [73]. Non-damaged remodeling cells are an active part of these futile programs, but the relative contribution of damage-induced remodeling cells as a cause of senescence (including late-life inflammation) remains an open question.

## References

[1] Campisi J (2013). Aging, cellular senescence, and cancer. Ann Rev Phys.

[2] Hayflick L, Moorhead PS (1961). The serial cultivation of human diploid stem cells. Exp Cell Res.

[3] Smith JR, Pereira-Smith OM (1996). Replicative senescence: implications for in vivo aging and tumor suppression. Science.

[4] Coppé J, Desprez P, Krtolica A, Campisi J (2010). The senescence-associated secretory phenotype: the dark side of tumor suppression. Annu Rev Pathol.

[5] Krtolica A, Parrinello S, Lockett S, Desprez P, Campisi J (2001). Senescent fibroblasts promote epithelial cell growth and tumorigenesis: a link between cancer and aging. Proc Natl Acad Sci U S A.

[6] Baker DJ, Childs BG, Durik M, Wijers ME, Sieben CJ, Zhong J, Saltness RA, Jeganathan KB, Verzosa GC, Pezeshki A (2016). Naturally occurring p16(Ink4a)-positive cells shorten healthy lifespan. Nature.

[7] Mylonas A, O’Loghlen A. Cellular senescence and ageing: mechanisms and interventions. Front Aging. 2022;3. 10.3389/fragi.2022.866718 PMC9261318 35821824

[8] Munoz-Espin D, Serrano M (2014). Cellular senescence: from physiology to pathology. Nat Rev Mol Cell Biol.

[9] Paramos-de-Carvalho D, Jacinto A, Saúde L (2021). The right time for senescence eLife.

[10] Saito Y, Chikenji TS (2021). Diverse roles of cellular senescence in skeletal muscle inflammation, regeneration, and therapeutics. Front Pharmacol.

[11] Yun MH (2018). Cellular senescence in tissue repair: every cloud has a silver lining. Int J Dev Biol.

[12] Elder SS, Emmerson E (2020). Senescent cells and macrophages: key players for regeneration?. Open Biol.

[13] Rhinn M, Ritschka B, Keyes WM (2019). Cellular senescence in development, regeneration and disease. Development.

[14] Harley CB, Futcher AB, Greider CW (1990). Telomeres shorten during ageing of human fibroblasts. Nature.

[15] Serrano M, Lin AW, McCurrach ME, Beach D, Lowe SW (1997). Oncogenic ras provokes premature cell senescence associated with accumulation of p53 and p16INK4a. Cell.

[16] Campisi J (1997). Aging and cancer: the double-edged sword of replicative senescence. J Am Geriatr Soc.

[17] Williams GC (1957). Pleiotropy, natural selection and the evolution of senescence. Evolution.

[18] Blagosklonny MV (2006). Aging and immortality: quasi-programmed senescence and its pharmacologic inhibition. Cell Cycle.

[19] de Magalhães JP, Church GM (2005). Genomes optimize reproduction: aging as a consequence of the developmental program. Physiology.

[20] Maklakov AA, Chapman T (2019). Evolution of ageing as a tangle of trade-offs: energy versus function. Proc Biol Sci.

[21] Gems D, de Magalhães JP (2021). The hoverfly and the wasp: A critique of the hallmarks of aging as a paradigm. Ageing Res Rev.

[22] Gems D (2022). The hyperfunction theory: an emerging paradigm for the biology of aging. Ageing Res Rev.

[23] Galimov ER, Lohr JN, Gems D (2019). When and how can death be an adaptation?. Biochem Mosc.

[24] Gems D, Kern CC. Biological constraint as a cause of aging. Preprints.org. 2022.

[25] Blagosklonny MV (2003). Cell senescence and hypermitogenic arrest. EMBO Rep.

[26] Nelson G, Wordsworth J, Wang C, Jurk D, Lawless C, Martin-Ruiz C, von Zglinicki T (2012). A senescent cell bystander effect: senescence-induced senescence. Aging Cell.

[27] Yousefzadeh M, Henpita C, Vyas R, Soto-Palma C, Robbins P, Niedernhofer L. DNA damage-how and why we age? eLife. 2021;10:e62852. 10.7554/eLife.62852 PMC7846274 33512317

[28] Baker DJ, Jeganathan KB, Cameron JD, Thompson M, Juneja S, Kopecka A, Kumar R, Jenkins RB, de Groen PC, Roche P (2004). BubR1 insufficiency causes early onset of aging-associated phenotypes and infertility in mice. Nat Genet.

[29] Yousefzadeh MJ, Flores RR, Zhu Y, Schmiechen ZC, Brooks RW, Trussoni CE, Cui Y, Angelini L, Lee KA, McGowan SJ (2021). An aged immune system drives senescence and ageing of solid organs. Nature.

[30] Schmitt CA, Fridman JS, Yang M, Lee S, Baranov E, Hoffman RM, Lowe SW (2002). A senescence program controlled by p53 and p16INK4a contributes to the outcome of cancer therapy. Cell.

[31] Wiley CD, Campisi J (2021). The metabolic roots of senescence: mechanisms and opportunities for intervention. Nat Metab.

[32] Plikus MV, Wang X, Sinha S, Forte E, Thompson SM, Herzog EL, Driskell RR, Rosenthal N, Biernaskie J, Horsley V (2021). Fibroblasts: Origins, definitions, and functions in health and disease. Cell.

[33] Parsonage G, Filer AD, Haworth O, Nash GB, Rainger GE, Salmon M, Buckley CD (2005). A stromal address code defined by fibroblasts. Trends Immunol.

[34] Demaria M, Ohtani N, Youssef S, Rodier F, Toussaint W, Mitchell J, Laberge R, Vijg J, Van Steeg H, Dollé M (2014). An essential role for senescent cells in optimal wound healing through secretion of PDGF-AA. Dev Cell.

[35] Storer M, Mas A, Robert-Moreno A, Pecoraro M, Ortells MC, Di Giacomo V, Yosef R, Pilpel N, Krizhanovsky V, Sharpe J (2013). Senescence is a developmental mechanism that contributes to embryonic growth and patterning. Cell.

[36] Menon R, Richardson LS, Lappas M (2019). Fetal membrane architecture, aging and inflammation in pregnancy and parturition. Placenta.

[37] Blagosklonny MV (2012). Answering the ultimate question "what is the proximal cause of aging?". Aging.

[38] Davan-Wetton CSA, Pessolano E, Perretti M, Montero-Melendez T (2021). Senescence under appraisal: hopes and challenges revisited. Cell Mol Life Sci.

[39] Medawar PB. An unsolved problem of biology. 1952.

[40] Krizhanovsky V, Yon M, Dickins RA, Hearn S, Simon J, Miething C, Yee H, Zender L, Lowe SW (2008). Senescence of activated stellate cells limits liver fibrosis. Cell.

[41] Hayflick L (1965). The limited in vitro lifetime of human diploid cell strains. Exp Cell Res.

[42] Coryell PR, Diekman BO, Loeser RF (2021). Mechanisms and therapeutic implications of cellular senescence in osteoarthritis. Nat Rev Rheumatol.

[43] Martin JA, Buckwalter JA (2003). The role of chondrocyte senescence in the pathogenesis of osteoarthritis and in limiting cartilage repair. J Bone Joint Surg Am.

[44] Diekman BO, Sessions GA, Collins JA, Knecht AK, Strum SL, Mitin NK, Carlson CS, Loeser RF, Sharpless NE (2018). Expression of p16 INK 4a is a biomarker of chondrocyte aging but does not cause osteoarthritis. Aging Cell.

[45] van der Kraan PM, van den Berg WB (2008). Osteoarthritis in the context of ageing and evolution. Loss of chondrocyte differentiation block during ageing. Ageing Res Rev.

[46] Yang X, Chen L, Xu X, Li C, Huang C, Deng CX (2001). TGF-beta/Smad3 signals repress chondrocyte hypertrophic differentiation and are required for maintaining articular cartilage. J Cell Biol.

[47] Ferguson CM, Schwarz EM, Reynolds PR, Puzas JE, Rosier RN, O’Keefe RJ (2000). Smad2 and 3 mediate transforming growth factor-beta1-induced inhibition of chondrocyte maturation. Endocrinology.

[48] Farnum CE, Lee R, O'Hara K, Urban JPG (2002). Volume increase in growth plate chondrocytes during hypertrophy: the contribution of organic osmolytes. Bone.

[49] Aigner T, Reichenberger E, Bertling W, Kirsch T, Stöss H, von der Mark K (1993). Type X collagen expression in osteoarthritic and rheumatoid articular cartilage. Virchows Arch B Cell Pathol Incl Mol Pathol.

[50] Hoyland JA, Thomas JT, Donn R, Marriott A, Ayad S, Boot-Handford RP, Grant ME, Freemont AJ (1991). Distribution of type X collagen mRNA in normal and osteoarthritic human cartilage. Bone Miner.

[51] Marsell R, Einhorn TA (2011). The biology of fracture healing. Injury.

[52] Blagosklonny MV (2021). The hyperfunction theory of aging: three common misconceptions. Oncoscience.

[53] Behmoaras J, Gil J (2021). Similarities and interplay between senescent cells and macrophages. J Cell Biol.

[54] Hall BM, Balan V, Gleiberman AS, Strom E, Krasnov P, Virtuoso LP, Rydkina E, Vujcic S, Balan K, Gitlin I (2016). Aging of mice is associated with p16(Ink4a)- and β-galactosidase- positive macrophage accumulation that can be induced in young mice by senescent cells. Aging (Albany NY).

[55] Baker D, Wijshake T, Tchkonia T, LeBrasseur N, Childs B, van de Sluis B, Kirkland J, van Deursen J (2011). Clearance of p16Ink4a-positive senescent cells delays ageing-associated disorders. Nature.

[56] Hall BM, Balan V, Gleiberman AS, Strom E, Krasnov P, Virtuoso LP, Rydkina E, Vujcic S, Balan K, Gitlin II (2017). p16(Ink4a) and senescence-associated β-galactosidase can be induced in macrophages as part of a reversible response to physiological stimuli. Aging (Albany NY).

[57] Serhan CN, Brain SD, Buckley CD, Gilroy DW, Haslett C, O’Neill LA, Perretti M, Rossi AG, Wallace JL (2007). Resolution of inflammation: state of the art, definitions and terms. FASEB J.

[58] Hu Y, Fryatt GL, Ghorbani M, Obst J, Menassa DA, Martin-Estebane M, Muntslag TAO, Olmos-Alonso A, Guerrero-Carrasco M, Thomas D (2021). Replicative senescence dictates the emergence of disease-associated microglia and contributes to Aβ pathology. Cell Rep.

[59] Talma N, Gerrits E, Wang B, Eggen BJL, Demaria M (2021). Identification of distinct and age-dependent p16 High microglia subtypes. Aging Cell.

[60] Guerrero A, De Strooper B, Arancibia-Cárcamo IL (2021). Cellular senescence at the crossroads of inflammation and Alzheimer's disease. Trends Neurosci.

[61] Gonzalez-Meljem JM, Martinez-Barbera JP (2021). Adamantinomatous craniopharyngioma as a model to understand paracrine and senescence-induced tumourigenesis. Cell Mol Life Sci.

[62] Apps JR, Carreno G, Gonzalez-Meljem JM, Haston S, Guiho R, Cooper JE, Manshaei S, Jani N, Hölsken A, Pettorini B (2018). Tumour compartment transcriptomics demonstrates the activation of inflammatory and odontogenic programmes in human adamantinomatous craniopharyngioma and identifies the MAPK/ERK pathway as a novel therapeutic target. Acta Neuropathol.

[63] Robbins PD, Jurk D, Khosla S, Kirkland JL, LeBrasseur NK, Miller JD, Passos JF, Pignolo RJ, Tchkonia T, Niedernhofer LJ (2021). Senolytic drugs: reducing senescent cell viability to extend health span. Annu Rev Pharmacol Toxicol.

[64] Jeon OH, Kim C, Laberge R-M, Demaria M, Rathod S, Vasserot AP, Chung JW, Kim DH, Poon Y, David N (2017). Local clearance of senescent cells attenuates the development of post-traumatic osteoarthritis and creates a pro-regenerative environment. Nat Med.

[65] Chen J, Long F (2014). mTORC1 signaling controls mammalian skeletal growth through stimulation of protein synthesis. Development.

[66] Blagosklonny MV (2012). Cell cycle arrest is not yet senescence, which is not just cell cycle arrest: terminology for TOR-driven aging. Aging (Albany NY).

[67] Pal B, Endisha H, Zhang Y, Kapoor M (2015). mTOR: a potential therapeutic target in osteoarthritis?. Drugs R D.

[68] Matsuzaki T, Matsushita T, Tabata Y, Saito T, Matsumoto T, Nagai K, Kuroda R, Kurosaka M (2014). Intra-articular administration of gelatin hydrogels incorporating rapamycin-micelles reduces the development of experimental osteoarthritis in a murine model. Biomaterials.

[69] Zhang Y, Vasheghani F, Li Y-H, Blati M, Simeone K, Fahmi H, Lussier B, Roughley P, Lagares L, Pelletier J-P (2015). Cartilage-specific deletion of mTOR upregulates autophagy and protects mice from osteoarthritis. Ann Rheum Dis.

[70] Takayama K, Kawakami Y, Kobayashi M, Greco N, Cummins JH, Matsushita T, Kuroda R, Kurosaka M, Fu FH, Huard J (2014). Local intra-articular injection of rapamycin delays articular cartilage degeneration in a murine model of osteoarthritis. Arthritis Res Ther.

[71] Carames B, Hasegawa A, Taniguchi N, Miyaki S, Blanco FJ, Lotz M (2012). Autophagy activation by rapamycin reduces severity of experimental osteoarthritis. Ann Rheum Dis.

[72] Post Y, Clevers H (2019). Defining adult stem cell function at its simplest: the ability to replace lost cells through mitosis. Cell Stem Cell.

[73] Friedman SL, Sheppard D, Duffield JS, Violette S (2013). Therapy for fibrotic diseases: nearing the starting line. Sci Transl Med.

